# Explaining decisions of graph convolutional neural networks: patient-specific molecular subnetworks responsible for metastasis prediction in breast cancer

**DOI:** 10.1186/s13073-021-00845-7

**Published:** 2021-03-11

**Authors:** Hryhorii Chereda, Annalen Bleckmann, Kerstin Menck, Júlia Perera-Bel, Philip Stegmaier, Florian Auer, Frank Kramer, Andreas Leha, Tim Beißbarth

**Affiliations:** 1 Medical Bioinformatics, University Medical Center Göttingen, Göttingen, Germany; 2 Dept. of Medicine A (Hematology, Oncology, Hemostaseology and Pulmonology), University Hospital Münster, Münster, Germany; 3 Hospital del Mar Medical Research Institute (IMIM), Barcelona, Spain; 4 geneXplain GmbH, Wolfenbüttel, Germany; 5 IT Infrastructure for Translational Medical Research, University of Augsburg, Augsburg, Germany; 6 Medical Statistics, University Medical Center Göttingen, Göttingen, Germany; 7 Campus-Institute Data Science (CIDAS), University of Göttingen, Göttingen, Germany

**Keywords:** Gene expression data, Explainable AI, Personalized medicine, Precision medicine, Classification of cancer, Deep learning, Prior knowledge, Molecular networks

## Abstract

**Background:**

Contemporary deep learning approaches show cutting-edge performance in a variety of complex prediction tasks. Nonetheless, the application of deep learning in healthcare remains limited since deep learning methods are often considered as non-interpretable black-box models. However, the machine learning community made recent elaborations on interpretability methods explaining data point-specific decisions of deep learning techniques. We believe that such explanations can assist the need in personalized precision medicine decisions via explaining patient-specific predictions.

**Methods:**

Layer-wise Relevance Propagation (LRP) is a technique to explain decisions of deep learning methods. It is widely used to interpret Convolutional Neural Networks (CNNs) applied on image data. Recently, CNNs started to extend towards non-Euclidean domains like graphs. Molecular networks are commonly represented as graphs detailing interactions between molecules. Gene expression data can be assigned to the vertices of these graphs. In other words, gene expression data can be structured by utilizing molecular network information as prior knowledge. Graph-CNNs can be applied to structured gene expression data, for example, to predict metastatic events in breast cancer. Therefore, there is a need for explanations showing which part of a molecular network is relevant for predicting an event, e.g., distant metastasis in cancer, for each individual patient.

**Results:**

We extended the procedure of LRP to make it available for Graph-CNN and tested its applicability on a large breast cancer dataset. We present Graph Layer-wise Relevance Propagation (GLRP) as a new method to explain the decisions made by Graph-CNNs. We demonstrate a sanity check of the developed GLRP on a hand-written digits dataset and then apply the method on gene expression data. We show that GLRP provides patient-specific molecular subnetworks that largely agree with clinical knowledge and identify common as well as novel, and potentially druggable, drivers of tumor progression.

**Conclusions:**

The developed method could be potentially highly useful on interpreting classification results in the context of different omics data and prior knowledge molecular networks on the individual patient level, as for example in precision medicine approaches or a molecular tumor board.

**Supplementary Information:**

The online version contains supplementary material available at (10.1186/s13073-021-00845-7).

## Background

Gene expression profiling by microarrays or next-generation sequencing has played a significant role in identifying predictive gene signatures and discovering individual biomarkers in cancer prognosis [1]. High-throughput sequencing produces huge amounts of gene expression data that can potentially be used for deriving clinical predictors (e.g., predicting occurrence of metastases) and identifying novel drug targets. Breast cancer is one of the paradigmatic examples of the utility of high-throughput data to derive prognostic molecular signatures (PAM50, MammaPrint, OncotypeDX) [2, 3] that predict clinical outcome. Based on the expression of 50 genes, the PAM50 classifier is widely used to divide breast cancers into four main molecular subtypes: luminal A, luminal B, triple-negative/basal-like, and HER2-enriched [4]. While the two luminal subtypes are characterized by high hormone receptor expression and generally have a better prognosis, the basal-like breast cancers are a heterogeneous group of hormone receptor- and HER2-negative breast cancers that are highly proliferative and often metastasize early. MammaPrint and OncotypeDX are 70- and 21-gene expression signatures that stratify patients according to the likelihood of metastasis. Although molecular signatures have prognostic impact, a more complete analysis of the molecular characteristics in the individual patient is required for personalized breast cancer therapy [2]. We hypothesize that molecular signatures can differ from one patient to another due to the heterogeneity of breast cancers. Such molecular signatures can be depicted as patient-specific subnetworks that are parts of a molecular network representing background knowledge about biological mechanisms. Presenting interpretable patient-specific subnetworks to clinicians and researchers enables better interpretability of the data for further medical and pharmaceutical insights, and possibly, for extended treatment options.

From a machine learning (ML) perspective, the prediction of a clinical outcome is a classification task, and molecular signatures can be identified as discriminative features. One drawback is that the search for molecular signatures is based on high-dimensional gene expression datasets, where the number of genes is much higher than the number of patients. The “curse of dimensionality” leads to instability in the feature selection process across different datasets. Stability can be improved including prior knowledge of molecular networks (e.g., pathways) into ML approaches [5]. ML methods benefit from pathway knowledge since neighboring genes are not treated as independent but instead similarities among adjacent genes, which should have similar expression profiles, are captured [6].

The essence of our classification task is to predict an occurrence of distant metastasis based on gene expression data structured by a molecular network (encoded as a graph) representing connections between genes. The patients are represented as graph signals (gene expression data) on a single graph. Since each vertex of a molecular network has a corresponding gene expression value as an attribute, we perform a graph signal classification task. Patients’ gene expression profiles create different graph signal patterns that can be learned by the means of deep learning.

In recent years, deep learning has been widely applied on image data using convolutional neural networks (CNNs). The CNNs exploit the grid-like structure of images and cannot directly process data structured in non-Euclidean domains. Examples of non-Euclidean data domains include networks in social sciences and molecular networks in biology. Recently, deep learning methods extended to domains like graphs and manifolds [7]. Graph-CNN [8] learns graph signal patterns and can be applied to our graph signal classification task.

Deep neural networks are able to model complex interactions between the input and output variables. This complexity does not allow to track what role a particular input feature plays in the output; thus, a neural network itself as a black-box ML model does not provide interpretable insights.

On the other hand, decisions proposed by neural networks have to be explained before they can be taken into account in the clinical domain [9]. The European Union’s recent General Data Protection Regulation (GDPR) restricted automated decision making produced by algorithms [10]. Article 13 of [10] specifies that clinics should provide patients with “meaningful information about the logic involved”. Article 22 of [10] states that a patient shall have the right not to be subject to an automated decision unless the patient gives a consent with it (paragraph 2.c). Therefore, the explainability of deep neural networks becomes an imperative for clinical applications.

Explanation methods aim at making classification decisions of complex ML models interpretable in terms of input variables. These methods use one of two available approaches [11]: functional or message passing. The first group of methods produces explanations out of local analysis of a prediction. It includes the sensitivity analysis, Taylor series expansion, and the model agnostic approaches LIME [12] and SHAP [13]. The second group [14, 15] provides explanations by running a backward pass in a computational graph, which generates a prediction as its output. The Layer-Wise Relevance Propagation (LRP) method [15] combines through the framework of deep Taylor decomposition [11] functional and message passing approaches to generate relevances of each input feature. For a fixed input feature, the relevance shows how much this feature influences the classifier’s decision. The relevances are generated for each data point (in our application each patient) individually.

In image data, LRP exhibited promising results and has been applied in cancer research to identify prognostic biomarkers: Klauschen et al. [16] applied LRP for visual scoring of tumor-infiltrating lymphocytes (TIL) on hematoxylin and eosin breast cancer images. Binder et al. [17] used LRP to identify spatial regions (cancer cell, stroma, TILs) on morphological tumor images that explained predictions of molecular tumor properties (like protein expression).

There are also some interpretation methods specialized for Graph Neural Networks (GNN). In [18–20], the authors provided explanation methods that are exclusively based on and crafted only for Graph Convolutional Network [21] utilizing a convolutional architecture which is a simplified version of that of Graph-CNN [8] we use. Ying et al. [22] suggested the model-agnostic GNNExplainer that is suitable for node classification, link prediction, and graph classification, but the authors did not consider an application of their approach to graph signal classification [23, 24], which is the problem at hand. The GNN-LRP method [25] proposes explanations in the form of scored sequences of edges on the input graph (i.e., relevant walks). Such a sequence represents a path extracted from the input to the output of GNN that brings insights for GNN’s decision strategy. This is useful especially for graph classification tasks, where each data point is represented as an individual graph. In our task, patients are represented as graph signals on a single graph, so that this method is not applicable.

Hence, there is still a lack of methods explaining individualized predictions in the context of graph signal classification task. Here, we adapted an existing LRP technique to graph convolutional layers of Graph-CNN [8] incorporating prior knowledge of a molecular network. Our approach generates explanations in the form of relevant subgraphs for each data point and allows to provide interpretable molecular subnetworks that are individual for each patient. According to the knowledge of the authors, an explanation method that benefits from prior knowledge and provides patient-specific subnetworks has not been shown before. The novelty of our work consists of two parts. First, we present the Graph Layer-wise Relevance Propagation (GLRP) method delivering data point-specific explanations for Graph-CNN [8]. Second, we train Graph-CNN on a large breast cancer dataset to predict an occurrence of distant metastasis and show how patient-specific molecular subnetworks assist in personalized precision medicine decisions: We interpret the classifier’s predictions by patient-specific subnetworks that explain the differential clinical outcome and identify therapeutic vulnerabilities.

## Methods

### Gene expression data and molecular network

#### Protein-protein interaction network

We used the Human Protein Reference Database (HPRD) protein-protein interaction (PPI) network [26] as the molecular network to structure the gene expression data. The database contains protein-protein interaction information based on yeast two-hybrid analysis, in vitro and in vivo methods. The PPI network is an undirected graph with binary interactions between pairs of proteins. The graph is not connected.

#### Breast cancer data

We applied our methods to a large breast cancer patient dataset that we previously studied and preprocessed [27]. That data is compiled out of 10 public microarray datasets measured on Affymetrix Human Genome HG-U133 Plus 2.0 and HG-U133A arrays. The datasets are available from the Gene Expression Omnibus (GEO) [28] data repository (accession numbers GSE25066, GSE20685, GSE19615, GSE17907, GSE16446, GSE17705, GSE2603, GSE11121, GSE7390, GSE6532). The RMA probe-summary algorithm [29] was used to process each of the datasets, and only samples with metadata on metastasis-free survival were selected and combined together on the basis of HG-U133A array probe names. Quantile normalization was applied over all datasets. In the case of several probes mapping to one gene, only the probe with the highest average value was considered. After pre-processing the dataset contained 12,179 genes in 969 patients. The patients were assigned to one of two classes: 393 patients with distant metastasis within the first 5 years and 576 patients without metastasis having the last follow-up between 5 and 10 years. Breast cancer molecular subtypes for the patient samples were predicted in [27] utilizing *genefu* R-package [30].

After mapping of 12,179 genes to the vertices of the PPI, the resulting PPI graph consisted of 7168 vertices (mapped genes) in 207 connected components. The main connected component had 6888 vertices, and each of the other 206 components had from 1 to 4 vertices. For further analyses, we utilized only the main connected component since the Graph-CNN requires the graph to be connected. The preprocessed data is provided in [31].

#### Expression data of HUVECs before and after TNF *α* stimulation

For validation purposes, we analyzed gene expression data from human umbilical vein endothelial cells (HUVECs) treated or not treated with tumor necrosis factor alpha TNF *α* [32]. The data, provided by the same authors (GEO database series: GSE144803), containing 39 sample pairs (treated and untreated), were suitable for a binary classification task and balanced. The expression data were quantile normalized and mapped to vertices of HPRD PPIs resulting in 7798 genes in the main connected component.

### Problem formulation

We focus on explaining classifier decisions of Graph-CNN adapting existing LRP approaches for graph convolutional layers. LRP should be applied as a postprocessing step to a model already trained for the ML task. The task is formulated as a binary classification of gene expression data *X*∈*R*^*n*×*m*^ to a target variable *Y*∈{0,1}^*n*^. *n* is the number of data points (patients) and *m* is the number of features (genes). The information of the molecular network is presented as an undirected weighted graph *G*=(*V*,*E*,*A*), where *V* and *E* denote the sets of vertices and edges respectively and *A* denotes the adjacency matrix. The Graph-CNN was designed to work with weighted graphs. We define weighted adjacency matrix *A* of dimensionality *m*×*m* since in general molecular networks can be weighted. For the unweighted HPRD PPI network, the matrix *A* has only “0s” and “1s” as its elements. A row *x* of the gene expression matrix *X* contains data from one data point (patient) and can be mapped to the vertices of the graph *G*. In such a way, values of *x* are interpreted as a graph signal.

A trained neural network can be represented as a function $f: R_{+}^{m} \to \left [0, 1\right ]$ mapping the input to the probability of the output class. The input *x* is a set of gene expression values *x*={*x*_*g*_} where *g* denotes a particular gene. The function *f*(*x*) computes the probability that a certain pattern of gene expression values is present w.r.t to the output class. LRP methods apply propagation rules from the output of the neural network to the input in order to quantify the relevance score *R*_*g*_(*x*) for each gene *g*. These relevances show how much gene *g* influences the prediction *f*(*x*) :

1 $$  \forall x : f(x) = \sum_{g} R_{g} (x).  $$

Equation (1) [11] demonstrates that the relevance scores are calculated w.r.t every input data point *x*.

### Graph Convolutional Neural Network and Layer-wise Relevance propagation

Usual CNNs learn data representations on grid-like structures. The Graph-CNN [8] as a deep learning technique is designed to learn features on weighted graphs. The convolution on graphs is used to capture localized patterns of a graph signal. This operation is based on spectral graph theory. The main operator to investigate the spectrum of a graph is the graph Laplacian *L*=*D*−*A*, where *D* is a weighted degree matrix, and *A* is a weighted adjacency matrix. *L* is a real symmetric positive semidefinite matrix that can be diagonalized such that *L*=*UΛU*^*T*^, where *Λ*=*diag*([*λ*_1_,…,*λ*_*m*_]) is a diagonal non-negative real valued matrix of eigenvalues, matrix *U* is composed of eigenvectors. Matrices *U* and *U*^*T*^ define the Fourier and the inverse Fourier transform respectively. According to the convolution theorem, the operation of graph convolution can be viewed as a filtering operation:

2 $$  y = h_{\theta}(L)x = h_{\theta}\left(U \Lambda U^{T}\right)x = U h_{\theta}(\Lambda)U^{T}x,  $$

where *x*,*y*∈*R*^*m*^, and the filter *h*_*θ*_(*Λ*) is a function of eigenvalues (graph frequencies). To localize filters in space, the authors in [8] decided to use a polynomial parametrization

3 $$  h_{\theta}(\Lambda) = \sum_{k=0}^{K-1}\theta_{k}\Lambda^{k},  $$

where *θ*∈*R*^*k*^ is a vector of parameters. The order of the polynomial, which is equal to *K*−1, specifies the local *K*−1 hop neighborhood. The neighborhood is determined by the shortest path distance. The polynomial filter can be computed recursively, as a Chebyshev expansion, which is commonly used in graph signal processing to approximate kernels [33]. The Chebyshev polynomial *T*_*k*_(*x*) of order *k* is calculated as *T*_*k*_(*x*)=2*xT*_*k*−1_(*x*)−*T*_*k*−2_(*x*) with *T*_0_=1 and *T*_1_=*x*. The Chebyshev expansion applies for values that lie in [−1,1]; therefore, the diagonal matrix of eigenvalues *Λ* has to be derived from a rescaled Laplacian *L*=(*D*−*A*)/*λ*_*max*_−*I*_*n*_. Thus, the filtering operation can be rewritten as

4 $$  y = h_{\theta}(\Lambda)x = \sum_{k = 0}^{K-1}\theta_{k} T_{k}(L)x = \left[\bar{x}_{0}, \ldots, \bar{x}_{K-1}\right] \theta,  $$

where $\bar {x}_{k} = 2L\bar {x}_{k-1}-\bar {x}_{k-2}$ with $\bar {x}_{0}=x$ and *x*_1_=*Lx*. The transition in Eq. 4 is done according to the observation (*UΛU*^*T*^)^*k*^=*UΛ*^*k*^*U*^*T*^. The filtering at the convolutional layer boils down to an efficient sequence of *K*−1 sparse matrix-vector multiplications and one dense matrix-vector multiplication [8].

LRP is based on the theoretical framework of deep Taylor decomposition. The function *f*(*x*) from Eq. (1) can be decomposed in terms of the Taylor expansion at some chosen root point *x*^∗^ so that *f*(*x*^∗^)=0. The first order Taylor expansion of f(x) is:

5 $$  \begin{aligned} f(x)& = f(x^{*}) + \sum_{g = 1}^{m} \frac{\partial f}{\partial x} |_{x = x^{*}} \cdot \left(x_{g} - x^{*}_{g}\right) + \epsilon\\ & = 0 + \sum_{g = 1}^{m} R_{g}(x) + \epsilon \end{aligned}  $$

where the relevances *R*_*g*_(*x*) are the partial differentials of the function *f*(*x*). The details of how to choose a good root point are described in [11]. The *f*(*x*) represents an output neuron of a neural network which consists of multiple layers and each layer consists of several neurons. A neuron receives a weighted sum of its inputs and applies a nonlinear activation function. The idea of the deep Taylor decomposition is to perform a first order Taylor expansion at each neuron of the neural network. These expansions allow to produce relevance propagation rules that compute relevances at each layer in a backward pass. The rules redistribute the relevance from layer to layer starting from output until the input is reached. The value of the output represents the model’s decision which is equal to the total relevance detected by the model.

LRP is commonly applied to deep neural networks consisting of layers with rectified linear units (ReLU) nonlinearities. In our experiments, we use only this activation function. Let *i* and *j* be single neurons at two consecutive layers at which the relevance should be propagated from *j* to *i*. The activation function has this form:

6 $$  a_{j} = max\left(0, \sum_{i} a_{i} w_{ij} + b_{j}\right)  $$

where *a*_*i*_,*a*_*j*_ are neurons’ values, *w*_*ij*_ are weights, and *b*_*j*_ is bias. Noticeably, the layers of this type always have non-negative activations. The relevance propagation rule is the following:

7 $$  R_{i} = \sum_{j} \frac{a_{i} w_{ij}^{+}}{\sum_{i} a_{i} w_{ij}^{+} + \epsilon} R_{j},  $$

where $w_{ij}^{+}$ corresponds to the positive weights *w*_*ij*_ and *ε* stabilizes numerical computations [9]. We set *ε* to 1^−10^. Equation (7) depicts the *z*^+^ rule coming from deep Taylor decomposition [11]. The *z*^+^ rule is commonly applied to the convolutional and fully connected layers. It favors the effect of only positive contributions to the model decisions. The first input layer can have other propagation rules that are specific to the domain [34]. In our work, we used the rule (7) for the input layer as well since the gene expression data has positive values.

In order to propagate relevance through the filtering (4), we rewrite it as follows:

8 $$  y = \sum_{k=0}^{K-1}\theta_{k} T_{k}(L) x = \left[\bar{L}_{0}, \ldots,\bar{L}_{K-1} \right] \theta x = W x,  $$

where matrix *W*∈*R*^*m*×*m*^ connects nodes *y* and *x*. The computation of matrix *W* is done as: $W=\left [\bar {L}_{0}, \ldots,\bar {L}_{K-1}\right ]\theta $, where $\bar {L}_{k} = 2L\bar {L}_{k-1}-\bar {L}_{k-2}$ with $\bar {L}_{0}=I$ and $\bar {L}_{1}=L$ are the Chebyshev polynomials of the Laplacian matrix.

Each convolutional layer has *F*_*in*_ channels

9 $$ \left[x_{1}, \ldots,x_{F_{in}}\right] \in R_{+}^{m \times F_{in}}  $$

in the input feature map and *F*_*out*_ channels

10 $$ \left[y_{1},\ldots, y_{F_{out}}\right] \in R^{m \times F_{out}}  $$

of the output feature map. We consider the values of output feature maps before applying ReLU non-linearities on them. The *F*_*in*_×*F*_*out*_ vectors of the Chebyshev coefficients *θ*_*i*,*j*_∈*R*^*k*^ are the layer’s trainable parameters. The input feature map can be transformed into a vector $\hat {x}=\left [x_{1}^{T}, \ldots, x_{F_{in}}^{T}\right ]^{T} \in R_{+}^{m \cdot F_{in}}$. We adapt Eq. (8) to compute the *j*^*th*^ channel of the output feature map based on the input feature map:

11 $$  \begin{aligned} y_{j}& = \left[\bar{L}_{0}, \ldots, \bar{L}_{K-1}\right] \cdot \left[\theta_{1,j}, \ldots, \theta_{F_{in}j}\right] \cdot \left[x_{1}^{T}, \ldots, x_{F_{in}}^{T}\right]^{T}\\ & = \left[\hat{L}_{1,j}, \ldots, \hat{L}_{F_{in},j}\right] \cdot \left[x_{1}^{T}, \ldots, x_{F_{in}}^{T} \right]^{T}\\ & = \hat{W}_{j} \times \hat{x} \in R^{m} \end{aligned}  $$

where $\hat {L}_{i,j} = \left [\bar {L}_{0}, \ldots, \bar {L}_{K-1}\right ] \theta _{i,j} \in R^{m \times m}, \hat {W}_{j} = \left [\hat {L}_{1,j}, \ldots, \hat {L}_{F_{in},j}\right ] \in R^{m \times m \cdot F_{in}}$

Since the *j*^*th*^ channel of the output feature map is connected through the matrix-vector multiplication with the input feature map, $\hat {W}_{j}$ can be treated as a matrix of weights joining two fully connected layers. Therefore, the relevance $R_{y}^{j} \in R_{+}^{m}$ from the *j*^*th*^ output channel can be propagated to the input feature map relevance $R_{\hat {x}}^{j} \in R_{+}^{m \cdot F_{in}}$ according to the rule (7). Overall, the relevance propagated from the output feature map to the input feature map is:

12 $$  R_{\hat{x}} = \sum_{j = 1}^{F_{out}} R_{\hat{x}}^{j} \in R_{+}^{m \cdot F_{in}}.  $$

For running LRP on graph convolutional layers, one needs to compute huge and dense matrices $\hat {W}_{j}$. It requires *K*−2 sparse matrix-matrix multiplications and one sparse to dense matrix-matrix multiplication. The computations for relevance propagation are heavier and much more memory demanding compared to the filtering (4). The code implementing our GLRP approach is available in [35].

### GLRP on gene expression data

To demonstrate the utility of GLRP, the Graph-CNNs were trained on two gene expression datasets described in the “Gene expression data and molecular network” section. In our previous study [23], the gene expression data were standardized for the training. But in this paper, we did not standardize the data. The argument for it is the following. For the non-image data, to standardize the input features is the usual practice. However, in case of standardization, the input features are treated independently. For an image, the neighboring pixels are highly correlated. If the pixels as features are standardized across the dataset, then this can distort the pattern of the image quite significantly and lead to misinterpretation. Analogically, feature wise standardization of microarray data changes expression patterns of genes located in the same neighborhood of a molecular network (HPRD PPI in our case). This might affect the explainability of the Graph-CNN that we aim at. Therefore, we trained the Graph-CNN directly on the quantile normalized data avoiding the additional standardization step. Instead, we subtracted the minimal value (5.84847) of the data from each cell of the gene expression matrix to keep the gene expression values non-negative. If initially, GE data was lying in [5.84847, 14.2014], now it is in the interval [0.0, 8.3529]. This transformation allows Graph-CNN to converge faster, to apply the LRP propagation rule (7) suitable for non-negative input values, and to preserve original gene expression patterns in local neighborhoods of the PPI network.

For each of the two gene expression datasets structured by the same prior knowledge (HPRD PPI), we used a 10-fold cross validation over a whole dataset to estimate the predictive performance of Graph-CNN. The hyperparameters such as the number of filters, the presence of pooling, the learning rate, and decay were tweaked manually on this 10-fold cross validation.

The architecture of the Graph-CNN trained on the HUVECs dataset and its performance are given in the “Comparison of subnetworks derived by GLRP to gene-coexpression networks identified by WGCNA” section.

For the breast cancer dataset, the Graph-CNN architecture consisted of two graph convolutional layers following maximum pooling of size 2, and two hidden fully connected layers with 512 and 128 units respectively. Each graph convolutional layer contained 32 filters covering the vertex’ neighborhood of size 7. For the performance comparison, we trained a “glmgraph” method [36] implementing network-constrained sparse regression model using HPRD PPI network, and Random Forest without any prior knowledge as baselines. The results on 10-fold cross validations are presented in the “GLRP to deliver patient-specific subnetworks” section.

**Fig. 1:**
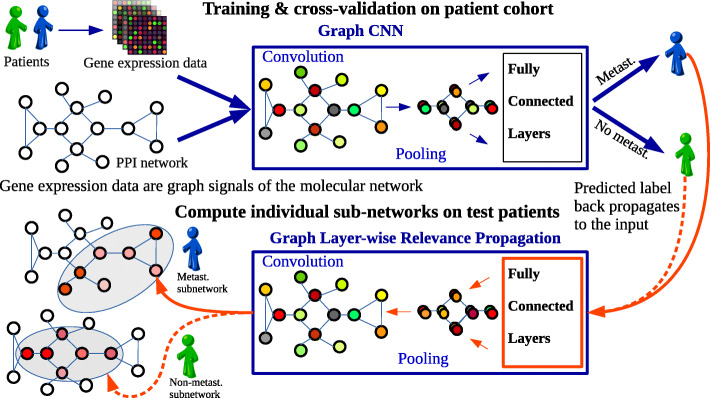
The workflow to obtain a data point-specific subnetwork. For clarity, a data point represented by a gene expression profile of a patient from the breast cancer dataset. The molecular network (HPRD PPI) structures the genes and is the same for every patient. Patient’s gene-expression values are assigned to every vertex of the HPRD PPI so that the patient is represented as a graph signal. Trained Graph-CNN performs graph convolutions and as output classifies the patient as metastatic or non-metastatic. GLRP is applied as a post hoc processing, propagating the relevance from the predicted label up to the input features (vertices of the molecular network). Top 140 highly relevant vertices constitute a molecular subnetwork. Molecular subnetworks differ from one patient to another

Further we generated the patient-specific (data point specific) subnetworks via GLRP. For that, each of the gene expression datasets was randomly split again: 90% training and 10% test. We retrained the Graph-CNN on 90% of data using manually selected hyperparameters from 10-fold cross validation, and propagated relevances on test data which was not “seen” by the model during training to make it more challenging. Since the LRP rule (7) propagates only positive contributions, our Graph-CNN had two output neurons for binary classification tasks that showed the probability of these two classes. For each patient in the test set, relevance was propagated by GLRP from the predicted output neuron to the input neurons representing genes (vertices) of the underlying molecular network. The workflow to deliver the patient-specific subnetworks is depicted on Fig. 1. A patient-specific subnetwork explaining the prediction was constructed from the 140 most relevant genes. Selecting more than 140 top relevant vertices entailed visualization issues. The singletons were deleted so that the subnetwork consisted mainly of around 130 vertices. The same workflow was applied to generate data-point-specific subnetworks for the data described in the “Expression data of HUVECs before and after TNF *α* stimulation” section.

### Pathway analysis

Enrichment of signal transduction pathways annotated in the TRANSPATH^*Ⓡ*^ database version 2020.1 [37] in genes prioritized by GLRP were analyzed using the geneXplain platform version 6.1 [38]. The analysis based on the Fisher’s exact test [39] was carried out for gene sets obtained for individual patients from the breast cancer dataset as well as for their combination into subtype gene sets.

The following calculations were applied to investigate differences in pathway hits. Let *P* denote a set of pathway genes and *S*_*i*_ and *S*_*k*_ two subnetwork gene sets, so that *P*_*i*_=*P*∩*S*_*i*_ and *P*_*k*_=*P*∩*S*_*k*_ are the sets of pathway genes matched by the two subnetworks. The difference △*P*_*i*,*k*_ in matched pathway genes was then calculated as |(*P*_*i*_∪*P*_*k*_)∖(*P*_*i*_∩*P*_*k*_)|/|*P*_*i*_∪*P*_*k*_| with |*P*_*i*_∪*P*_*k*_|>0. For each selected pathway, we calculated △*P*_*i*_,*k* for each pair of subnetworks and reported the median of examined pairs.

### Comparison of subnetworks derived by GLRP to gene-coexpression networks identified by WGCNA

To further examine the biological relevance of subnetwork genes prioritized by GLRP and for the purpose of comparison to an already available method that uses expression and network information to prioritize gene sets, we analyzed the gene expression data described in “Expression data of HUVECs before and after TNF *α* stimulation” section. We compared gene sets identified in our subnetworks to gene modules and differentially expressed genes in response to TNF *α* identified by Rhead et al. [32]. Rhead et al. [32] reported gene modules obtained by weighted gene co-expression network analysis (WGCNA). The method has been applied in many studies and constructs a gene network based on expression measurements from which it can derive modules of co-expressed genes [40]. We trained a Graph-CNN on the gene expression data to classify the TNF *α* treatment status of HUVECs. The Graph-CNN architecture consisted of 2 convolutional layers with 4 and 8 filters respectively followed by one hidden fully connected layer with 128 nodes. The vertex’s neighborhood covered by graph convolutions was of size 7. No pooling was used. The performance of the Graph-CNN in 10-fold cross validation: mean 100*AUC, accuracy, and F1-weighted were 99.49, 96.25% and 96.06%, respectively. A random forest achieved the same performance. We generated the subnetworks according to the “GLRP on gene expression data” section, retrained the Graph-CNN on 70 randomly selected samples, and applied GLRP on 8 test samples (4 treated and 4 not treated). The test samples were predicted correctly. For each of the 8 test samples, we constructed a subnetwork. Associations between subnetwork genes sets and 16 gene modules defined by Rhead et al. [32] as well as 589 upregulated genes (log-fold change > 0.5, FDR < 0.01), 425 downregulated genes (log-fold change < − 0.5, FDR < 0.01), and the combined set of 1014 DE genes were analyzed using the *Functional classification* tool of the geneXplain platform [41]. Fisher test calculations were carried out with a total contingency table count corresponding to the number of genes in [32, file S1 of] after mapping to Ensembl [42] gene ids (10022 genes). Rhead et al. [32] assigned a color code to the 16 gene co-expression modules and denoted them as *black*, *blue*, *brown*, *cyan*, *green*, *greenyellow*, *grey*, *magenta*, *midnightblue*, *pink*, *purple*, *red*, *salmon*, *tan*, *turquoise*, and *yellow* which is maintained in results reported here.

## Results

### Sanity check of the implemented graph LRP

To initially validate our implemented LRP, we applied Graph-CNN on the MNIST dataset [43] in the same way as described in the paper [8]. The MNIST dataset contains 70,000 images of hand-written digits each having a size of 28 by 28 pixels. To apply Graph-CNN on the image data, we constructed an 8 nearest-neighbors graph similarly to the schema proposed in [8], with the exception that all the weights are equal to 1. The weight 1 is more natural for the graph connecting neighboring image pixels. Thus, each image is a graph signal represented by node attributes—pixel values. We achieved high classification accuracy (99.02%) on the test set for the Graph-CNN, which is comparable to the performance of classical CNN (99.33%) reported in [8]. The number of parameters was the same for both methods.

**Fig. 2:**
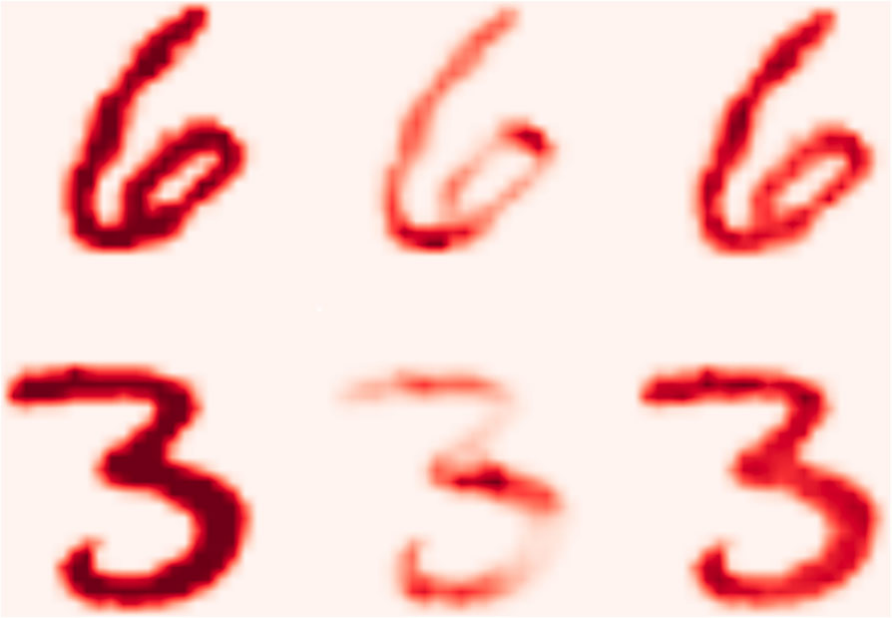
From left to right: initial image, LRP on classical CNN and GLRP on Graph-CNN

Usually, to manage box-constrained pixel values, the special pixel-specific LRP rule is applied for the input layer. This pixel-specific rule highlights not only the digits itself, but also the contours of the digits [34, Fig. 13 of]. In contrast, the rule (7) highlights only those positively relevant parts of the image where the signal of the digit is present. We kept the propagation rule (7) for the input and all other layers in all our experiments. Further, we visually compared on the same digits how the heatmaps generated by implemented GLRP correspond to the heatmaps generated by usual LRP procedure applied on classical CNN (Fig. 2).

The heatmaps were rendered only for the classes predicted by classical CNN and Graph-CNN. In this case, the classes are “6” and “3”. For the Graph-CNN, a bigger part of the digit is relevant for the classification since the covered neighborhood can be expanded up to 24 hops. Graph-CNN’s filters are isotropic; thus, they tend to cover roundish areas that concern rounded patterns (curves) of the digit (Additional file 1: Fig. S1).

### Genes selected by GLRP correlate with modules identified by gene co-expression network analysis

In the analysis of TNF-induced gene expression changes in HUVECs, our procedure prioritized in total 168 genes of which 105 genes were found in subnetworks of all eight test samples (Additional file 2). Remarkably, the *green* gene module, which was the most strongly correlated one with TNF *α* upregulation [32], showed significant association (adjusted *p* value < 0.05) with the combined set of subnetwork genes, with genes found in the majority of subnetworks and also with 5 of the 8 subnetworks (Additional file 2). At the same significance level, the *turquoise* gene module described in [32] was strongly associated with 2 of 8 subnetworks and with genes found in all 8 subnetworks. In addition, both the *green* and the *turquoise* modules showed moderate association (adjusted *p* value < 0.1) with the majority of gene sets defined on the basis of the test subnetworks. Furthermore, we found strong (adjusted *p* value < 0.05) or moderately (adjusted *p* value < 0.1) significant overlap between upregulated genes and some subnetwork gene sets. The gene modules *cyan*, *greenyellow*, and *midnightblue* did not overlap with GLRP-derived subnetworks. These results demonstrate partial agreement between gene sets suggested by GLRP, another gene network analysis and classical differential expression analysis. Hence, the GLRP-based subnetworks gathered biologically meaningful genes and may even complement other approaches in revealing important properties of the underlying biological systems. Additionally, another two gene sets were compared with WGCNA modules: the intersection of subnetworks genes and genes that occurred in more than in 4 test samples subnetworks. Notably, the individual subnetworks shared more genes with the *green* and *turquoise* WGCNA modules than those described gene sets, pointing out the ability of GLRP to identify sample-specific genes.

### GLRP to deliver patient-specific subnetworks

We applied the GLRP to the Graph-CNN trained on gene expression data from the “Breast cancer data” section. The gene expression data was structured by a protein-protein interaction network. The standardization of features was not performed as described in the “GLRP on gene expression data” section. The prediction task performed by the Graph-CNN was to classify patients into 2 groups, metastatic and non-metastatic. The results of a 10-fold cross validation are depicted in Table 1. While Graph-CNN and glmgraph utilized the HPRD PPI network topology, a random forest did not use any prior knowledge. glmgraph was not evaluated on non-standardized data, since it had convergence issues in this case. The metrics were averaged over folds and the standard errors of their means were calculated.

**Table 1:** Performance of Graph-CNN on metastatic event prediction, depending on normalization

Method	Std	100*AUC	Accuracy, %	F1-weighted, %
Graph-CNN	-	82.57 ±1.25	76.07 ±1.30	75.82 ±1.33
Random Forest	-	81.27 ±1.66	74.23 ±1.73	73.47 ±1.84
Graph-CNN	+	82.16 ±1.25	76.18 ±1.36	75.86 ±1.35
Random Forest	+	81.40 ±1.76	74.74 ±1.67	74.00 ±1.82
glmgraph	+	80.88 ±1.37	75.14 ±1.30	74.73 ±1.39

Std stands for standardization of features (genes)

The GLRP was applied as described in the“GLRP on gene expression data” section. We retrained the Graph-CNN on 872 patients and generated relevances for 97 test patients. The relevances were propagated from the Graph-CNN’s output node corresponding to the correctly predicted class. The most frequently selected features are summarized in Additional file 1: Table S1. The eukaryotic translation elongation factor EEF1A1, which is overexpressed in the majority of breast cancers and protects tumor cells from proteotoxic stress [44], was the sole factor that was selected in all of the 97 test set patients. Other frequently selected features in both non-metastatic as well as metastatic patients included genes such as the epithelial-to-mesenchymal-transition (EMT)-related gene VIM (46/58 non-metastatic, 30/39 metastatic patients), the extracellular matrix protein FN1 (43/58 non-metastatic, 22/39 metastatic patients), the actin cytoskeleton regulator CFL1 (7/58 non-metastatic, 7/39 metastatic patients), and the estrogen receptor ESR1 28/58 non-metastatic, 10/39 metastatic patients) that are all known to be linked with breast cancer development and progression [45–48]. This indicates that our method successfully identified relevant key players with a general role in breast tumorigenesis.

Additionally, we show individualized PPI subnetworks delivered for four correctly predicted breast cancer patients (Table 2) from the microarray data set. Two of them had been assigned with the most common subtype luminal A (LumA), while the other two suffered from the highly aggressive basal-like subtype. In each group, one patient with early metastasis was picked and one who did not develop any within at least 5 years of follow-up.

**Table 2:** Patients that the PPI subnetworks are generated for

Patient’s ID	Subtype	Metastatic event	Time of metastases, years	Last follow-up, years
GSM519217	Basal	1	0.9	-
GSM615233	LumA	1	0.79	-
GSM615695	Basal	0	-	5.38
GSM150990	LumA	0	-	9.93

**Fig. 3:**
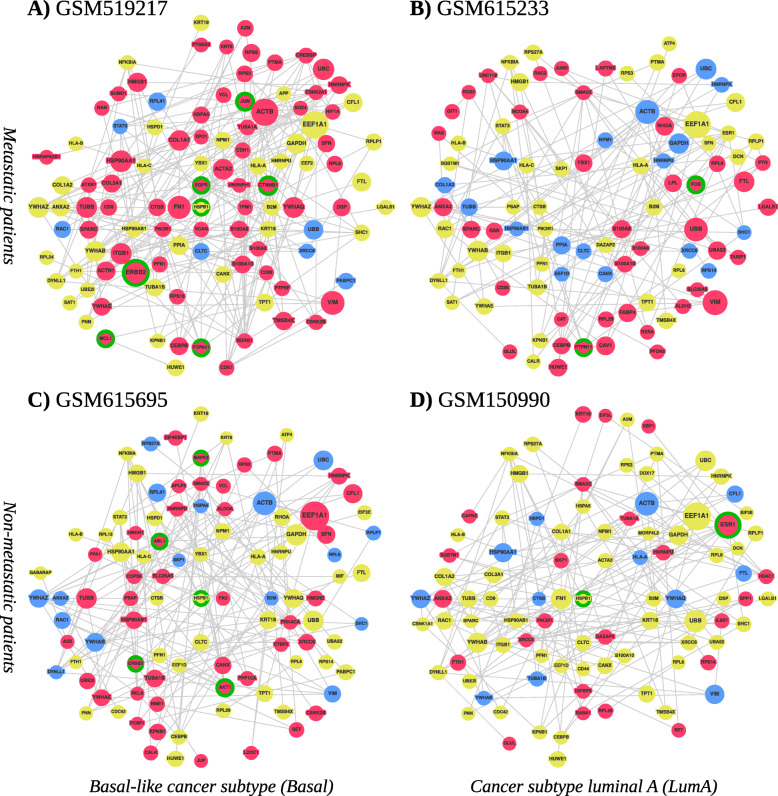
The PPI subnetworks for (1) metastatic patients **a** (GSM519217) and **b** (GSM615233) and (2) non-metastatic patients **c** (GSM615695) and **d** (GSM150990). The coloring of the node is based on gene expression levels by 25% and 75% quantiles (blue=LOW, yellow=NORMAL, red=HIGH), based on the gene expression throughout the whole patient cohort. The size of vertices corresponds to the relevance scores within one subnetwork. All the subnetworks are highly relevant compared to the rest of the PPI network. Green circles highlight targetable genes

The generated PPI subnetworks are displayed in Fig. 3. The sequence of pictures in order ABCD is the same as in the table.

Interestingly, the networks of both LumA patients contained ESR1 which fits well since this subtype is considered as estrogen receptor positive [49]. In contrast, genes often associated with the basal-like subtype and a poor prognosis such as MCL1, CTNNB1, EGFR, or SOX4 were found in the basal-like patient GSM519217 suggesting that the generated networks are capable of extracting breast cancer subtype-specific features. The comparison of the subnetworks of the non-metastatic and the metastatic patients furthermore revealed some patient-specific genes which might give valuable information about specific mechanisms of tumorigenesis and therapeutic vulnerabilities in the respective patient. In general, it seemed that the subnetworks of the non-metastatic patients contained more genes that have been linked to better prognostic outcomes such as JUP, PCBP1, and HMGN2 in GSM615695 [50–52] or RASA1, IL6ST, KRT19, and RPS14 in GSM150990 [53–56], while the networks of both metastatic patients harbored genes that are known to be involved in aggressive tumor growth or therapy resistance which might explain the early metastatic spread in these patients. Some examples are CDK1, SFN, and XPO1 in GSM519217 [57–59] or CAV1, PTPN11, and FTL in GSM615233 [60–62].

However, not only the presence of specific genes might be important, but also their overall expression level. Our analyses identified, e.g., the EMT-related gene VIM as one of the most relevant nodes in the subnetworks of both metastatic patients in which the gene was highly expressed (> 75% quantile based on the gene expression throughout the whole patient cohort). In contrast, VIM was also present in the subnetworks of the two non-metastatic patients, however, with a lower relevance and a particularly low expression (< 25% quantile). VIM is an important marker for EMT and high expression levels correlate with a motile, mesenchymal-like cancer cell state, thus making VIM an essential effector of metastasis [45].

**Fig. 4:**
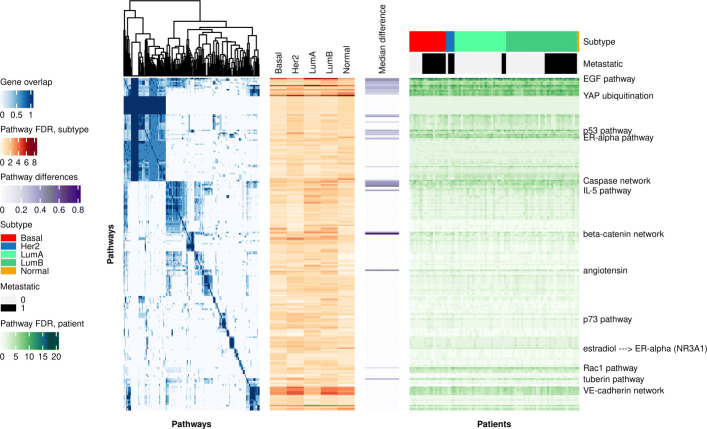
Signal transduction pathway analysis of subnetwork genes reported for 79 patients in 5 subtypes. (From left to right) Blue heatmap: 238 signaling pathways clustered according to proportion of shared subnetwork genes; Orange heatmap: Enrichment significance of pathways in subnetwork genes combined from patients of given subtype. Darker orange indicates higher significance; Purple heatmap: Median difference in matched pathway genes observed in pairwise comparisons of subnetwork gene sets from patients mapped to 33 pathways. Darker purple indicates higher tendency of pairs of subnetwork gene sets to coincide with different pathway genes; Green heatmap: Enrichment significance of pathways in subnetwork genes of 79 patients. Darker green indicates higher significance. Corresponding subtypes and metastatic status are shown by the annotation above the heatmap. A detailed version of this figure capturing pathway and sample names is provided in Additional file 1: Fig. S2

A comparison of subnetwork genes of 79 correctly predicted test set patients to a database of signal transduction pathways confirmed significant enrichment of pathways that have previously been associated with cancer disease mechanisms such as the EGF, ER-alpha, p53, and TGFbeta pathways as well as Caspase and beta-catenin networks. Comparisons were performed for each patient as well as for subtype gene sets formed by combining subnetwork genes of patients associated with a breast cancer subtype. Results for the 238 signaling pathways from the TRANSPATH^*Ⓡ*^ database that were significantly enriched with subtype genes are visualized in Fig. 4. Differences in enrichment significance may suggest that the importance of some signaling pathways detected this way is subtype-specific, e.g., for YAP ubiquitination or the VE-cadherin network (orange heatmap, Fig. 4, see also Additional file 1: Table S2 for details). The pattern of enrichment found on the level of cancer subtypes coincided well with the findings for subnetwork genes of individual patients revealing several molecular networks with elevated significance in both subtype and patient gene sets such as the EGF pathway, although the patient-level visualization did not suggest subtype-specific enrichment (green heatmap, Fig. 4). One source of these observations can be that patient subnetworks tend to be associated with certain pathways but cover different pathway components (genes). We therefore compared pathway genes in pairs of patient subnetworks for the 33 largest pathways. In 18 pathways, the median pair of patient subnetworks differed in 33% or more of the genes matched within a pathway (see also Additional file 1: Table S3 for details). These results demonstrate that the subnetworks obtained by Graph-CNN were enriched with common signaling pathways relevant for the respective disease and can assign patient-specific priorities to pathway components.

Finally, we tested whether the subnetworks can also be used for finding potentially targetable genetic vulnerabilities that could open new options for personalized treatment decisions. We applied the “MTB report” methodology described in [63] to identify actionable genes present in the subnetworks. For that, we extended the algorithm to match high expression with gain of function alterations, and low expression with loss of function alterations. The results are summarized in Table 3.

**Table 3:** Actionable genes identified by the MTB report workflow

Patient	Gene	Expression	Known Var	Predicts
615695	HSPB1	Normal	expression	Response to gemcitabine
	ABL1	High	GoF	Response to ABL TK inhibitors (imatinib, desatininb, ponatinib, regorafenib …)
	AKT1	High	GoF	Response to PI3K, AKT, MTOR inhibitors; resistance to BRAF inhibitors
	ERBB2	High	GoF	Response to ERBB2, EGFR, MTOR, AKT inhibitors
	MAPK3	High	GoF	Resistance to EGFR inhibition
519217	HSPB1	Normal	expression	Response to gemcitabine
	CTNNB1	High	GoF	Response to everolimus + letrozole; resistance to Tankyrase inhibitors
	EGFR	High	GoF	Response to EGFR, ERBB2, HSP90 and MEK inhibitors
	ERBB2	High	GoF	Response to ERBB2, EGFR, MTOR, AKT inhibitors
	JUN	High	overexpr	Response to irbesartan (angiotensin II antagonist)
	MCL1	High	GoF	Resistance to anti-tubulin agents
	PTPN11	High	GoF	Response to MEK inhibitors
615233	FOS	High	overexpr	Response to irbesartan (angiotensin II antagonist)
	PTPN11	High	GoF	Response to MEK inhibitors
150990	HSPB1	Normal	expression	Response to gemcitabine
	ESR1	High	GoF	Response to novel ER degraders, fulvestrant, tamoxifen

Genes from the PPI subnetworks were matched to known genomic alterations (Known Var) that predict either response or resistance to drugs (Predicts). High and low gene expression were matched to gain of function (GoF) and loss of function (LoF) genomic variants, respectively

Although information about the presence of actionable genetic variants is missing from our patient microarray data, the information generated by the PPI subnetworks could be used to define specific panels for subsequent sequencing. Indeed, the MTB reports highlighted specific genes that could be targeted therapeutically in each of the four patients: In the non-metastatic LumA patient GSM150990 ESR1 was proposed as therapeutic target which is in line with current treatment regimens that use hormone therapy as the main first-line treatment of choice for this patient subgroup. In contrast, in the metastatic LumA patient GSM615233 FOS and PTPN11 were identified as novel actionable alterations. In the often rapidly relapsing basal-like patients HSPB1 and ERBB2 were identified as common targets as well as MAPK3, AKT1, and ABL1 for the non-metastatic patient GSM615695 or EGFR, MCL1, CTNNB1, PTPN11, and JUN for the metastatic patient GSM519217, thereby suggesting novel possibilities for combinatory or alternative treatments. Taken together, GLRP provides subnetworks centered around known oncogenic drivers that seem reasonable in the context of cancer biology and can help to identify patient-specific cancer dependencies and therapeutic vulnerabilities in the context of precision oncology.

## Discussion

In our work, we focused on the interpretability of a deep learning method utilizing molecular networks as prior knowledge. We implemented LRP for Graph-CNN and provided the sanity check of the developed approach on the MNIST dataset. Essentially, the main aim of the paper was to explain the prediction of metastasis for breast cancer patients by providing an individual molecular subnetwork specific for each patient. The patient-specific subnetworks provided interpretability of the deep learning method and demonstrated clinically relevant results on the breast cancer dataset.

Supposedly, the performance of Graph-CNN can be improved. The batch normalization technique [64] that is used to accelerate the training of deep neural networks is not seen to be available for the Graph-CNN, so this can be the way to enhance its performance. The LRP rule for batch normalization layers is yet another procedure to be adapted for Graph-CNN.

Another possibility to identify genes (and construct subnetworks out of them) influencing classifier decisions is to apply model-agnostic SHAP and LIME explanation methods. LIME method provides explanations of a data point based on feature perturbations. The method samples perturbations from a Gaussian distribution, ignoring correlations between features. It leads to the instability of explanations that is not favorable for personalized medicine. SHAP provides Shapley values for each feature of a data point as well but does not have such an issue, so we attempted to derive patients-specific subnetworks applying TreeExplainer and KernelExplainer from SHAP python module on Random Forest and Graph-CNN respectively. The subnetworks were build on the basis of HPRD PPI utilizing positive Shapley values, which were pushing prediction to a higher probability of corresponding class (metastatic or non-metastatic). The subnetworks obtained were mostly consisting from single vertices. In contrast, the subnetworks from GLRP and Graph-CNN were mostly connected. The SHAP’s DeepExplainer approach suitable for convenient deep learning models is not applicable for Graph-CNN. The model-agnostic KernelExplainer computes SHAP values out of a debiased lasso regression. Reevaluating the model happens several thousands numbers of times specified by a user as well as a small background dataset is needed for integrating out features. Hence, the KernelExplainer is not scalable and application of it on Graph-CNN resulted in not connected subnetworks as well.

Furthermore, the sensitivity of Graph-CNN to the changes of prior knowledge is still to be investigated. Authors in [8] showed that for the MNIST images a random graph connecting pixels significantly decreases the performance destroying local connectivity. In our case, the permutation of the vertices of the PPI network does not influence the classifier performance on standardized gene expression data. Yet, PPI network is a small world network and its degree distribution fits to the power law with the exponent *α*=2.70. It implies great connectivity between proteins and means that any two nodes are separated by less than six hops. The filters of convolutional layers cover a 7-hop neighborhood of each vertex, so we assume it still might be enough to capture the gene expression patterns. In our future work, we will investigate how the properties of the prior knowledge influence the performance and explainability of Graph-CNN.

The subnetworks generated by GLRP contained common potential oncogenic drivers which indicates that they can extract the essential cancer pathways. Indeed, our analyses identified genes associated with hormone receptor-positive breast cancer (e.g. ESR1, IL6ST, CD36, GLUL, RASA1) in the networks from the patients with estrogen receptor positive, LumA breast cancer and genes associated with the basal-like subtype (e.g., EGFR, SOX4, AKT1 as well as high levels of HNRNPK) in the basal-like patients, underlining the biological relevance of the networks. Next to subtype-specific genes, the networks contained several oncogenes that were found in all four patients and could thus represent common drivers of breast cancer initiation and progression. One example is the actin-binding protein cofilin (CFL1) that regulates cancer cell motility and invasiveness [46]. Another interesting candidate is STAT3 which is activated in more than 40% of breast cancers and can cause deregulated cell proliferation and epithelial-to-mesenchymal transition (EMT) [65]. Our graphs not only displayed patient-specific PPI subnetworks, but also concisely visualized the relevance of each node and its expression levels. This information is potentially relevant to judge the biological significance of the gene in a patient-specific context.

Next to the common genes found in all four networks, each network was characterized by several special, cancer-associated genes which are of high interest because they might represent patient-specific central signaling nodes and therapeutic vulnerabilities. Some examples are PTPN11 that is known to activate a transcriptional program associated with cancer stem cells or the EMT-related genes SOX4 or VIM that might be responsible for the high invasive capacity of the tumors and their early metastasis formation [45, 61, 66, 67]. Interestingly, the network of the metastatic patient GSM615233 harbored the genes FABP4 and LPL which both have been shown to interact with CD36, another highly expressed node in the network, to support cell proliferation and counteract apoptosis [68–70]. In contrast, in the non-metastatic patient GSM150990 especially the interleukin receptor IL6ST and the Ras GTPase-activating protein 1 (RASA1) seem to be interesting because for both high expression levels have been linked with a favorable prognosis [53, 54]. In the other non-metastatic patient GSM615695 high levels of HMGN2 and PCBP1 were identified which both have been shown to be able to inhibit cell proliferation [51, 52]. Although the experimental validation for the networks is still missing, it is tempting to speculate that these genes might contribute to the benign phenotype of the tumor in these patients.

All patient-specific subnetworks contained relevant drug targets that have been largely studied in breast cancer (e.g., ERBB2, ESR1, EGFR, AKT1). Yet, resistance mechanisms in breast cancer targeted therapies represent a big challenge; many of the identified therapeutic approaches have failed [71] due to the highly interconnected nature of signaling pathways and potential circumvents. A promising way forward could involve the molecular characterization of the tumor with transcriptomics and a parallel culture of patient-derived organoids. PPI networks could elucidate the right combination strategy by identifying central signaling nodes. Different therapeutic strategies could be tested on organoids and confirm the best strategy that synergistically blocks cancer cell escape routes and minimizes the emergence of survival mechanisms. Only the identification of relevant mechanisms of action for cell survival as well as of the factors involved in resistance for each patient, together with a more precise and personalized characterization of each cancer phenotype, may provide useful improvements in current therapeutic approaches.

## Conclusions

We present a novel Graph-CNN-based feature selection method that benefits from prior knowledge and provides patient-specific subnetworks. We adapted the existing Layer-wise Relevance Propagation technique to the Graph-CNN, demonstrated it on MNIST data, and showed its applicability on a large breast cancer dataset. Our new approach generated individual patient-specific molecular subnetworks that influenced the model’s decision in the given context of a classification problem. The subnetworks selected by the developed method utilizing general prior knowledge are relevant for prediction of metastasis in breast cancer. They contain common as well as subtype-specific cancer genes that match the clinical subtype of the patients, together with patient-specific genes that could potentially be linked to aggressive/benign phenotypes. In the context of a breast cancer dataset GLRP provides patient-specific explanations for the Graph-CNN that largely agree with clinical knowledge, include oncogenic drivers of tumor progression, and can help to identify therapeutic vulnerabilities. We therefore conclude that our method GLRP in combination with Graph-CNN is a new, useful, and interpretable ML approach for high-dimensional genomic data-sets. Generated classifiers rely on prior knowledge of molecular networks and can be interpreted by patient-specific subnetworks driving the individual classification result. These subnetworks can be visualized and interpreted in a biomedical context on the individual patient level. This approach could thus be useful for precision medicine approaches such as for example the molecular tumorboard.

## Supplementary Information

**Additional file 1** Contains Supplementary Tables S1-S3 and Supplementary Figures S1, S2.

**Additional file 2** Worksheet *Subnetwork genes 8 samples* provides identifiers and gene symbols of 167 subnetwork genes, in how many and in which samples they were selected. Worksheet *Gene module enrichment* presents results of Fisher test calculations comparing subnetwork gene sets to gene modules and DE gene sets. Each row contains data for a DE gene set or a gene module consisting of the total group size and column tripletts with *p*-value, adjusted *p*-value as well as the number of hits, respectively, observed in comparisons to the union of genes from 8 subnetworks, the set of genes occurring in the majority, the set of genes found in all of the subnetworks and each of the 8 samples. Highlighted are rows corresponding to *green* and *turquoise* gene modules, which were most often significantly associated with subnetwork gene sets (grey), adjusted *p*-values below 0.05 (red) and between 0.05 and 0.1 (yellow).

## Data Availability

The utilized breast cancer datasets are accessible from Gene Expression Omnibus (GEO) [28] data repository (accession numbers GSE25066, GSE20685, GSE19615, GSE17907, GSE16446, GSE17705, GSE2603, GSE11121, GSE7390, GSE6532). The HUVECs gene expression data [32] is available in GEO database (GSE144803). The HPRD PPI network can be found in [26]. The preprocessed breast cancer data, the adjacency matrix of the HPRD PPI network, and the code of the GLRP method are provided in http://mypathsem.bioinf.med.uni-goettingen.de/resources/glrp[31] and https://gitlab.gwdg.de/UKEBpublic/graph-lrp[35]. The web-site to explore patient-specific subnetworks is in http://mypathsem.bioinf.med.unigoettingen.de/MetaRelSubNetVis/[72].

## Abbreviations

ML: Machine learning
LRP: Layer-wise Relevance Propagation
GNN: Graph Neural Network
CNN: Convolutional Neural Network
GLRP: Graph Layer-wise Relevance Propagation
WGCNA: Weighted gene co-expression network analysis
GDPR: General Data Protection Regulation
GEO: Gene Expression Omnibus
HPRD: Human Protein Reference Database
PPI: Protein-protein interaction
ReLU: Rectified linear unit
HUVEC: Human umbilical vein endothelial cells
EMT: Epithelial-to-mesenchymal transition
