# Supplementary material for: Explaining decisions of graph convolutional neural networks: patient-specific molecular subnetworks responsible for metastasis prediction in breast cancer
Source: Genome Med. 2021 Mar 11;13:42. doi: 10.1186/s13073-021-00845-7 (PMC7953710; doi:10.1186/s13073-021-00845-7)
Supplement: Supplementary file 1 — Additional file 1 Contains Supplementary Tables S1-S3 and Supplementary Figures S1, S2. [file 13073_2021_845_MOESM1_ESM.pdf]

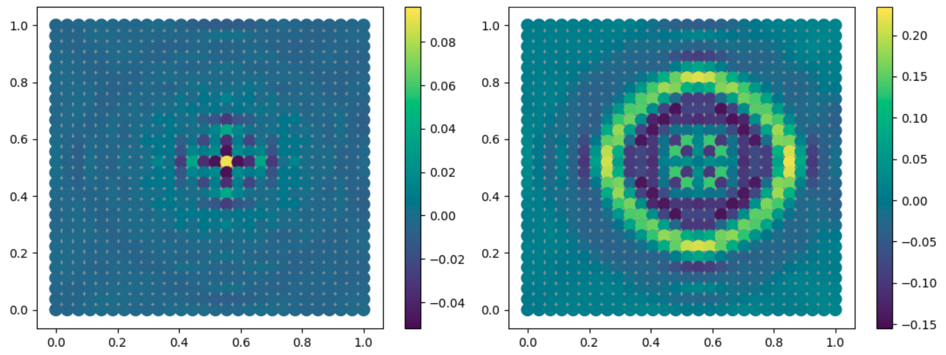

Supplementary Figure S1

Figure 1. Visualization of 2 out of 32 learned filters of the 1st convolutional layer of graph CNN classifying MNIST digits on the 8-nearest-neighbours graph. The convolutional filters have 25 parameters each. This means that the neighborhood covered by convolution can be expanded up to 24 hops from the vertice where the filter is centered. Due to rotational invariance, filters tend to learn patterns that concern rounded curves on the 8-nearest-neighbours graph. PyGSP[1] package was used for visualization.

[1] Defferrard, M., Martin, L., Pena, R., and Perraudin, N. PyGSP: Graph Signal Processing in Python. DOI: 10.5281/zenodo.1003157

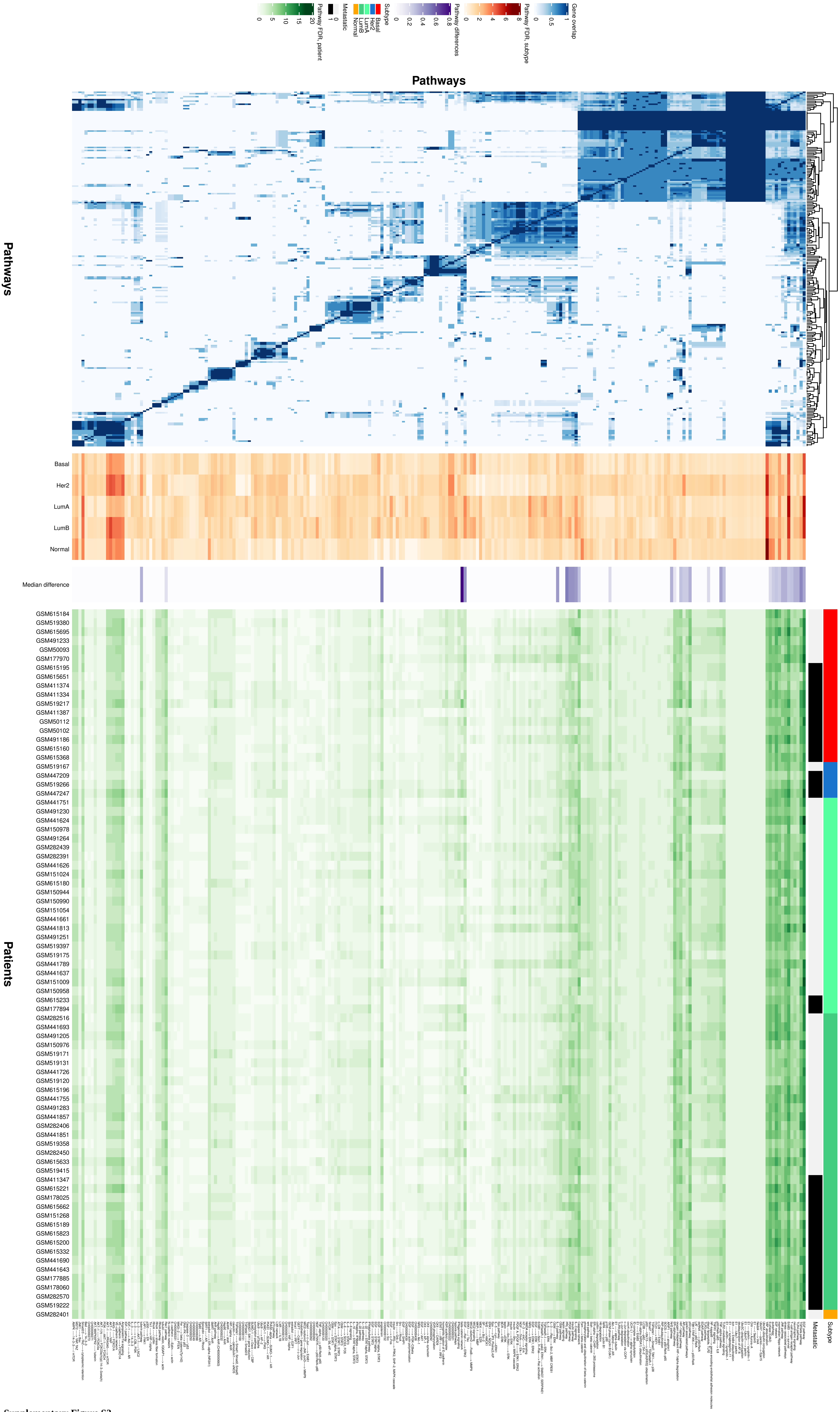

Supplementary Figure S2

Figure 2. Signal transduction pathway analysis of subnetwork genes reported for 79 correctly classified test set patients in 5 subtypes. This image resembles the corresponding figure of the main text while diplaying also pathway and sample names. The visualization was created using the ComplexHeatmap R package [2]. (From left to right) Blue heatmap: 238 signaling pathways clustered according to proportion of shared subnetwork genes; Orange heatmap: Enrichment significance of pathways in subnetwork genes combined from patients of given subtype. Darker orange indicates higher significance; Purple heatmap: Median difference in matched pathway genes observed in pairwise comparisons of subnetwork gene sets from patients mapped to 33 pathways. Darker purple indicates higher tendency of pairs of subnetwork gene sets to coincide with different pathway genes; Green heatmap: Enrichment significance of pathways in subnetwork genes of 79 patients. Darker green indicates higher significance. Corresponding subtypes and metastatic status are shown by the annotation above the heatmap.

[2] Gu, Z., Eils, R., Schlesner, M. (2016). Complex heatmaps reveal patterns and correlations in multidimensional genomic data, Bioinformatics , 32(18), 2847–2849, https://doi.org/10.1093/bioinformatics/btw313 .

Supplementary Table S1

Table 1. Frequency of gene selection in top 10 of highly relevant genes among metastatic and non-metastatic patients.

Metastatic (39 patients)		Non-metastatic (58 patients)	
**Gene**	**Frequency**	**Gene**	**Frequency**
EEF1A1	39	EEF1A1	58
UBC	39	ACTB	57
ACTB	39	UBB	57
UBB	38	UBC	56
GAPDH	33	HSP90AA1	46
HSP90AA1	31	VIM	46
VIM	30	GAPDH	44
YWHAQ	29	FN1	43
YWHAZ	29	YWHAB	42
YWHAB	27	YWHAQ	30
FN1	22	ESR1	28
ESR1	10	YWHAZ	25
TUBB	8	RPL41	18
FTL	7	TUBB	10
CFL1	7	COL1A2	9
RPL41	6	COL1A1	9
PPIA	5	FTL	8
ERBB2	5	CFL1	7
COL1A1	4	CLTC	7
KRT18	4	KRT18	7

Supplementary Table S2

Table 2. 238 signal transduction pathways from the TRANSPATHⓇ database that were significantly enriched (FDR < 0.05) in subnetwork genes associated with 5 cancer subtypes. The table shows pathway accessions and names as well as the -$\log_{10}$(adjusted p-value) of Fisher's exact test which was used to determine enrichment of pathway genes in subtype gene sets. Adjustment of p-values was carried out using the Benjamini-Hochberg method[2]. Transformation of values was performed to support display in heatmaps. The sum was calculated to sort pathways by their overall importance.

Pathway	Name	Basal	Her2	LumA	LumB	Normal	Sum
CH000004693	YAP ubiquitination	4.174	5.528	4.38	5.153	6.821	26.056
CH000004191	ErbB3 ---> survival	4.692	4.437	5.925	5.171	3.916	24.141
CH000000722	EGF pathway	4.692	3.649	5.851	5.453	2.944	22.588
CH000004208	AKT-1 ---/YAP	3.513	4.617	2.712	4.827	3.916	19.584
CH000004646	Smad2/3 ---TAZ---> cytoplasmic retention	3.091	3.206	4.38	4.005	3.589	18.271
CH000004615	ANG-1 ---/ FOXO1A	3.091	4.437	2.472	3.943	3.098	17.041
CH000004598	VE-cadherin network	3.093	3.726	2.647	3.943	3.57	16.978
CH000004597	VE-cadherin ---> FOXO1A	3.093	3.726	2.647	3.943	3.57	16.978
CH000004613	Angiopoietin/Tie signaling	3.006	4.437	2.472	3.64	3.357	16.912
CH000004539	AKT-1 ---PRAS40---> mTOR	3.006	4.086	2.285	3.902	3.57	16.849
CH000004600	mammalian Hippo network	3.025	2.893	3.142	2.854	3.647	15.561
CH000000692	S phase (Cdk2)	2.302	2.695	2.053	2.661	3.57	13.281
CH000000321	CH000000321	2.725	3.532	2.718	2.668	1.42	13.063
CH000000322	CH000000322	2.725	3.532	2.718	2.668	1.42	13.063
CH000004475	IL-3 signaling	2.409	1.767	3.017	3.236	2.292	12.721
CH000004599	MST2 ---> YAP, TAZ	1.89	2.695	2.033	2.632	3.355	12.604
CH000004572	EGF ---Raf-1---> ERK2	2.789	1.639	3.496	3.236	0.938	12.1
CH000004526	autophagy	1.77	2.414	1.584	2.603	3.35	11.72
CH000000580	Fam ---> beta-catenin	2.074	1.488	2.285	2.147	3.57	11.563
CH000000711	TGFbeta pathway	1.995	1.099	2.647	3.065	1.962	10.767
CH000000743	tuberin pathway	2.406	3.148	2.053	1.838	1.279	10.724
CH000000756	OSM pathway	1.259	1.639	2.891	2.665	1.872	10.326
CH000004192	NRG ---> ERK	1.944	1.347	3.311	2.537	1.085	10.224
CH000004538	AMPK ---14-3-3---/ mTOR	1.526	2.2	1.621	2.026	2.804	10.177
CH000000626	EGF ---> STAT1alpha, STAT3	2.725	2.2	1.791	2.668	0.765	10.149
CH000003550	sumoylation pathway	1.794	2.434	1.925	2.661	1.179	9.993
CH000000232	CH000000232	1.89	2.414	2.018	1.962	1.487	9.77
CH000004506	IL-5 pathway	2.14	1.639	2.366	2.147	1.392	9.685
CH000004243	estradiol ---> ER-alpha (NR3A1)	1.187	1.675	1.925	1.832	2.95	9.569
CH000000879	Caspase network	1.989	1.828	1.596	2.661	1.487	9.561
CH000004625	MIC2 signaling	3.006	0.866	1.621	2.884	1.002	9.379
CH000000736	PDGF pathway	1.89	1.512	1.721	3.003	1.179	9.304
CH000000956	E1 ---/ alpha-tubulin	1.522	2.127	1.621	1.49	2.481	9.24
CH000004667	MEK ---> EZR	1.526	0.62	2.472	2.374	2.217	9.21
CH000000987	hypoxia pathways	1.372	1.042	2.033	3.08	1.664	9.19
CH000004634	TGFbetaR-I ---> ERK	1.693	0.617	2.975	2.171	1.539	8.995
CH000000869	p73 pathway	1.921	2.185	1.596	1.962	1.279	8.942
CH000004649	AT1A ---> STAT1, STAT3	1.357	1.871	2.285	2.147	1.279	8.938

[2] Benjamini, Y., & Hochberg, Y. (1995). Controlling the False Discovery Rate: A Practical and Powerful Approach to Multiple Testing. *Journal of the Royal Statistical Society. Series B (Methodological), 57*(1), 289-300.

CH000004583	homophilic ligation of E-cadherin	1.906	1.767	2.053	1.965	1.211	8.902
CH000001012	ARIP1 ---> atrophin1	1.921	1.635	2.093	1.962	1.279	8.889
CH000000755	TLR4 pathway	1.666	1.899	1.508	2.276	1.487	8.836
CH000000123	CH000000123	1.693	2.2	1.791	1.729	1.42	8.834
CH000000124	CH000000124	1.693	2.2	1.791	1.729	1.42	8.834
CH000001016	AR pathway	1.187	3.251	1.654	1.43	1.111	8.633
CH000004590	tubulin ---restin, IQGAP1---> actin	1.315	2.165	1.508	1.729	1.909	8.626
CH000000999	EGF ---> c-Fos	1.522	0.866	3.005	2.147	1.002	8.542
CH000000721	SMRT ---/ AhR	1.794	2.434	1.925	1.2	1.179	8.532
CH000000851	JNK pathway	1.121	2.695	1.288	1.867	1.487	8.458
CH000003912	neurotrophic signaling	1.581	0.962	1.791	2.388	1.586	8.307
CH000000030	EGF ---> ERK1, ERK2	1.574	0.9	2.399	2.264	1.058	8.195
CH000004510	IL-5 ---> ERK	1.234	1.447	1.791	2.171	1.539	8.183
CH000004533	STAT1 ---IRF-1---> IRF2	1.693	2.2	1.791	1.729	0.765	8.179
CH000000724	EGF ---> ERK	1.526	1.114	2.216	2.026	1.279	8.161
CH000000719	AhR pathway	1.992	2.2	1.621	1.49	0.831	8.134
CH000000926	PRL pathway	1.89	1.365	1.68	2.171	0.914	8.019
CH000003878	SOCS-1 ---> p50:RelA-p65	1.526	1.995	1.621	1.514	1.328	7.985
CH000004521	TLR2-mediated signaling	1.023	2.342	1.171	2.232	1.179	7.947
CH000000715	HIF-1alpha pathway	1.526	1.709	1.459	2.026	1.179	7.899
CH000000747	E2F network	1.77	1.675	1.038	1.884	1.529	7.896
CH000004623	MIC2-isoform2 ---FosB---> MMP9	2.693	0.516	1.291	2.661	0.626	7.787
CH000000718	HIF-1alpha ---/ AhR	1.794	1.675	1.291	1.832	1.179	7.771
CH000000583	Siah ---> beta-catenin	1.12	1.767	1.243	1.49	2.111	7.731
CH000004471	IFNgamma signaling	1.061	2.2	1.584	1.399	1.42	7.664
CH000000839	c-Jun degradation via COP1	1.239	1.767	1.375	1.292	1.909	7.58
CH000004341	ER-alpha pathway	0.807	1.639	1.14	1.162	2.804	7.553
CH000000750	insulin pathway	1.526	1.393	2.033	1.399	1.179	7.53
CH000000770	beta-catenin network	1.855	1.531	1.776	1.729	0.615	7.505
CH000000874	ERK1 ---/ Tau	1.234	0.767	2.366	2.298	0.837	7.503
CH000003547	PIAS2-alpha ---SUMO-1---/ AR	0.991	2.2	1.791	1.729	0.765	7.476
CH000004149	Nrf2 pathway	1.006	1.365	1.508	2.068	1.51	7.456
CH000000758	stress-associated pathways	1.186	1.773	1.338	1.492	1.632	7.421
CH000000045	Hsp90 ---> AhR	1.357	1.871	1.508	1.399	1.279	7.414
CH000000909	Ubc9 ---> AP-2	1.52	1.871	1.596	1.514	0.904	7.404
CH000004666	NF-kappaB ---> genes encoding endothelial adhesion molecules	0.991	2.165	1.086	1.729	1.42	7.391
CH000000997	Ubc9 ---/ p73alpha	1.526	1.995	1.621	1.514	0.73	7.387
CH000004484	KSR scaffold complex	1.466	1.635	1.596	1.407	1.279	7.383
CH000000603	Epo ---> STAT1alpha, STAT3	1.693	1.365	1.791	1.729	0.765	7.343
CH000003552	SUMO-1 ---> Daxx ---/ CBP	1.89	1.488	1.171	1.962	0.792	7.303
CH000004455	caveolin-1 ---calmodulin1---> eNOS	1.693	1.635	1.186	1.122	1.644	7.28
CH000004722	cxcr4 ubiquitination	1.693	1.365	1.065	1.729	1.42	7.272
CH000000952	UCH-L1 ---> alpha-synuclein	1.079	1.488	1.171	1.962	1.487	7.186
CH000000624	EGF ---/ AKT	1.239	1.767	1.375	1.965	0.655	7
CH000004624	MIC2-isoform2 ---JNK, JunD---> MMP9	2.074	1.157	0.851	2.147	0.695	6.924
CH000004477	IL-3 ---> 14-3-3	1.22	1.257	1.813	1.217	1.41	6.918
CH000004247	ER-alpha (NR3A1) ubiquitination	0.814	1.157	1.508	1.399	2.039	6.917
CH000000573	phosphorylation and ubiquitination of beta-catenin	1.061	1.257	1.171	1.399	2.014	6.903

CH000000576	AKT, MAPKAPK2 ---/ tuberin	1.693	1.773	1.334	1.217	0.868	6.886
CH000000663	Hsp90 ---> AhR (CH000000663)	1.239	1.767	1.375	1.292	1.211	6.883
CH000000725	c-Cbl ---/ ErbB1	1.239	1.767	1.375	1.292	1.211	6.883
CH000000761	IL-10 pathway	0.814	1.871	1.508	1.399	1.279	6.87
CH000000584	IL-22 ---> STAT1alpha, STAT3	0.814	1.871	1.508	1.399	1.279	6.87
CH000000762	IL-22 pathway	0.814	1.871	1.508	1.399	1.279	6.87
CH000000579	IL-10 ---> STAT1alpha, STAT3	0.814	1.871	1.508	1.399	1.279	6.87
CH000000567	OSM ---> ERK	0.747	0.829	2.033	1.832	1.42	6.86
CH000000991	CKII ---> AP-1	0.991	1.365	1.065	2.668	0.765	6.854
CH000000821	TBK1:TRIF:IKK-i ---> p50:RelA	1.103	1.675	1.208	1.52	1.328	6.835
CH000004642	TGFbetaR-I ---pak2, ERK1---> SMAD7, SERPINE1	1.248	0.623	2.472	1.292	1.179	6.814
CH000004303	NGF ---p75NTR---> p50:RelA-p65	0.991	0.866	1.068	2.884	1.002	6.811
CH000000568	OSM ---> STAT3	0.498	1.365	1.791	1.729	1.42	6.803
CH000004534	IL-6 ---> SOCS3, FOS	0.498	1.365	1.791	1.729	1.42	6.803
CH000004483	IL-3 ---Ras---> Bcl-2, MBP, CREB1	0.807	0.866	1.644	1.962	1.487	6.766
CH000003548	PIAS1 ---SUMO-1---/ AR	0.903	1.995	1.621	1.514	0.73	6.763
CH000004690	LXR ---/ IL1B	1.693	2.2	1.791	0.986	0	6.671
CH000000824	dsRNA ---> p50:RelA	1.075	1.642	1.171	1.49	1.279	6.657
CH000000331	CH000000331	1.693	0.666	1.791	1.729	0.765	6.644
CH000000729	EGF ---> STAT3	1.693	1.365	1.065	1.729	0.765	6.617
CH000004525	diacyl lipopeptide, TLR2	0.693	1.675	1.068	1.965	1.179	6.58
CH000000820	TLR3 pathway	0.991	1.705	1.093	1.288	1.487	6.563
CH000001010	ITCH ---> HEF1	0.991	1.365	1.791	0.986	1.42	6.552
CH000000720	AhRR ---/ AhR	1.187	1.675	1.291	1.2	1.179	6.532
CH000000728	EGF ---> STAT1alpha	1.89	1.488	1.171	1.962	0	6.51
CH000004595	E-cadherin ---Eplin---> actin	1.187	1.021	1.291	1.832	1.179	6.51
CH000003574	SIRT1 ---/ AR	0.814	1.871	2.285	0.814	0.695	6.478
CH000000026	IL-6 ---> STAT3	0.589	1.642	1.334	1.292	1.586	6.443
CH000000759	EDAR pathway	0.991	1.642	1.086	1.3	1.42	6.439
CH000004536	IL-6 signaling	0.754	1.085	1.375	1.965	1.211	6.39
CH000004438	Ubc5C ubiquitin conjugation	1.079	1.488	1.171	1.122	1.487	6.347
CH000003545	Chfr ---> Aurora-A	1.079	1.488	1.171	1.122	1.487	6.347
CH000000001	RhoA ---/ cofilin	1.079	1.488	1.171	1.122	1.487	6.347
CH000000581	deubiquitination of AF-6	1.079	1.488	1.171	1.122	1.487	6.347
CH000004439	Ubc5A ubiquitin conjugation	1.079	1.488	1.171	1.122	1.487	6.347
CH000000751	T-cell antigen receptor pathway	1.009	1.085	1.429	1.399	1.42	6.342
CH000001027	TopoI ---> c-Jun ---> ErbB1	1.234	1.642	1.334	1.292	0.837	6.339
CH000004474	brca1 ---> IRF7	1.234	1.642	1.334	1.292	0.837	6.339
CH000000752	Rac1 pathway	1.079	1.675	0.952	0.986	1.644	6.336
CH000004632	Smad7 ---SIK---/ TGFbetaR-I	0.814	1.157	1.508	0.814	2.039	6.332
CH000004692	LXR network	1.357	1.871	2.285	0.814	0	6.327
CH000000590	LAT ---> p50:RelA	0.991	1.559	1.068	1.383	1.235	6.235
CH000004481	IL-3 ---AKT-1---/ LNC2	0.628	1.512	1.502	1.793	0.792	6.228
CH000004501	leptin signaling	1.327	1.099	1.171	1.616	1.012	6.224
CH000000439	CH000000439	0.903	1.257	2.472	1.514	0	6.146
CH000004670	oxygen independent HIF-1alpha degradation	0.991	1.767	0.799	1.182	1.315	6.053
CH000004726	ID complex deubiquitylation	1.357	1.157	0.851	1.399	1.279	6.044
CH000004570	ErbB1 ---Sos1, H-Ras---> Rac activation	1.181	0.886	1.715	1.514	0.742	6.039

CH000004494	IFNalpha, IFNbeta ---> STAT3	0.689	1.675	1.291	1.2	1.179	6.034
CH000004467	p50:RelA-p65 ---> IL8	0.991	1.767	1.068	0.884	1.315	6.024
CH000001011	MAGI-2 ---> PTEN	1.526	0.62	1.621	1.514	0.73	6.012
CH000004294	NT-3 ---trkC---> Erk	1.013	0.682	1.596	1.407	1.279	5.976
CH000004576	p53 ---Ubc13---> p53{ub{K63}} ubiquitination	1.526	1.257	0.952	0.877	1.328	5.94
CH000000951	CKII ---> alpha-synuclein	0.991	1.365	1.065	1.729	0.765	5.915
CH000004446	TNFR1 signaling	0.773	1.767	0.839	1.318	1.211	5.907
CH000000772	TNF-alpha pathway	0.923	1.286	0.815	1.492	1.371	5.887
CH000000878	Src ---/ RhoA	1.312	1.365	0.954	1.327	0.912	5.87
CH000003888	EGF ---> Src	1.693	1.365	1.065	1.729	0	5.852
CH000004175	E1 ---UbcH7---> parkin	0.991	1.365	1.065	0.986	1.42	5.826
CH000004178	E1 ---Ubc7---> parkin	0.991	1.365	1.065	0.986	1.42	5.826
CH000004696	itch ---> ErbB4 ubiquitination	0.991	1.365	1.065	0.986	1.42	5.826
CH000000120	CH000000120	0.991	1.365	1.065	0.986	1.42	5.826
CH000003901	Nedd4 ---> trkA	0.991	1.365	1.065	0.986	1.42	5.826
CH000004502	leptin --->ERK	0.991	0.666	1.529	1.383	1.235	5.803
CH000000741	Epo pathway	1.259	0.767	1.456	1.292	0.938	5.712
CH000000152	CH000000152	1.079	1.488	2.018	1.122	0	5.707
CH000001018	Tip60 ---> AR	0.589	1.642	1.334	1.292	0.837	5.695
CH000003825	Epicardin ---/ Rhox5	0.589	1.642	1.334	1.292	0.837	5.695
CH000004188	NRG ---> Akt-1	0.975	1.512	1.502	1.318	0.377	5.684
CH000004578	cofilin-1 degradation	0.975	1.512	0.664	1.318	1.211	5.68
CH000004252	NGF ---TrkA---> Elk-1	0.975	0.651	1.502	1.318	1.211	5.657
CH000000732	EGF ---> SHIP1	1.079	1.488	1.171	1.122	0.792	5.652
CH000000710	p53 pathway	1.234	1.668	0.613	1.319	0.815	5.649
CH000000271	CH000000271	1.234	1.642	0.613	1.292	0.837	5.618
CH000000081	AKT-1(h) ---> FOXO3a(h){p}:14-3-3zeta(h)	1.234	1.642	0.613	1.292	0.837	5.618
CH000000459	LAP1 ---> FAK1	1.234	1.642	0.613	1.292	0.837	5.618
CH000000273	CH000000273	1.234	1.642	0.613	1.292	0.837	5.618
CH000000566	OSM ---> STAT1	0.45	1.257	1.621	1.514	0.73	5.571
CH000004517	TLR9 pathway	0.654	1.157	1.065	1.292	1.392	5.561
CH000000656	insulin ---Shc---> MAPK cascade	0.916	0.762	1.776	1.2	0.904	5.557
CH000004479	RIP deubiquitination	0.814	1.157	0.851	1.399	1.279	5.5
CH000000694	G2/M phase (cyclin B:Cdk1)	0.991	1.512	0.613	1.182	1.179	5.475
CH000000537	GEFT ---Cdc42---> c-Jun	1.234	0.767	1.334	1.292	0.837	5.464
CH000004540	LC3B ---p62---/ Dvl-2	0.991	0.866	1.068	0.986	1.508	5.419
CH000004720	Endothelin-1 gene regulation	0.498	0.666	1.791	0.986	1.42	5.361
CH000004679	Slim ---/ HIF-1alpha	1.526	0.62	0.952	1.514	0.73	5.344
CH000004482	Bad ---> 14-3-3	1.526	1.257	0.952	0.877	0.73	5.343
CH000004176	UbcH7 ---parkin---> Eps15	0.903	1.257	0.952	0.877	1.328	5.317
CH000000834	JNK1 ---> Bax	0.903	1.257	0.952	0.877	1.328	5.317
CH000000958	E1 ---/ Septin5	0.903	1.257	0.952	0.877	1.328	5.317
CH000000960	E1 ---parkin---/ synphilin-1	0.903	1.257	0.952	0.877	1.328	5.317
CH000000959	E1 ---/ synaptotagmin-11	0.903	1.257	0.952	0.877	1.328	5.317
CH000000962	E1 ---dorfin---/ synphilin-1	0.903	1.257	0.952	0.877	1.328	5.317
CH000000731	EGF ---> PI3K-C2beta	0.903	1.257	1.621	1.514	0	5.294
CH000000632	PDGF A, PDGF B ---> ERK1, ERK2	0.958	0.41	1.531	1.399	0.946	5.244
CH000004630	TGFbeta1 ---> Smad2/3	0.814	0.579	2.285	0.814	0.695	5.186

CH000000707	DNA-PK ---> p53	1.693	0.666	1.065	0.986	0.765	5.175
CH000004559	CKII ---/ CARD9	0.991	1.365	1.065	0.986	0.765	5.171
CH000000292	CH000000292	0.991	1.365	1.065	0.986	0.765	5.171
CH000004393	VDR phosphorylation	0.991	1.365	1.065	0.986	0.765	5.171
CH000003551	SUMO-1 ---> PIAS	0.991	1.365	1.065	0.986	0.765	5.171
CH000000961	E1 ---/ ErbB3	0.754	1.085	1.375	0.725	1.211	5.15
CH000004648	angiotensin	0.811	0.622	1.148	1.386	1.179	5.145
CH000004656	ERK1 ---> NQO1	0.991	0.666	1.065	0.986	1.42	5.127
CH000000574	mTOR ----> S6, eIF-4E	0.991	1.488	1.068	0.986	0.556	5.088
CH000000967	PI3K ---AKT-1---/ FOXO4	1.079	1.488	0.558	1.122	0.792	5.039
CH000004213	AKT-1 ---/ FOXO3a	1.079	1.488	0.558	1.122	0.792	5.039
CH000004283	ER-alpha ---CHIP---> 26S proteasome	0.539	1.257	0.804	1.01	1.42	5.028
CH000003412	Akt-1 ---Mdm2---> AR	0.589	1.871	0.613	1.184	0.765	5.021
CH000000749	RhoA pathway	1.181	1.488	0.636	0.814	0.883	5.003
CH000000662	RhoA ---> stress fiber formation	1.181	1.488	0.636	0.814	0.883	5.003
CH000000889	Fer ---> beta-catenin{Tyr142}	0.814	0.579	1.508	1.399	0.695	4.995
CH000000604	Epo ---> IP3, PtsIns(3,4)P	0.814	0.579	1.508	1.399	0.695	4.995
CH000000947	parkin associated pathways	0.773	1.512	0.613	0.911	1.179	4.986
CH000004593	N-cadherin network	0.991	0.435	1.068	1.49	1.002	4.986
CH000004512	IL-5 ---Lyn--->ERK1	0.903	0.617	1.369	0.848	1.179	4.916
CH000000547	Src ---> Grb-2	1.234	0.767	0.613	2.298	0	4.912
CH000000629	TNF-alpha ---> p50:RelA-p65	0.903	0.792	0.954	1.327	0.912	4.887
CH000000769	wnt pathway	0.689	0.983	0.752	0.855	1.577	4.857
CH000000121	p105 ---> p50	0.573	1.365	0.613	1.14	1.105	4.796
CH000000708	Caspase-3 ---/ p53	1.526	0.62	0.952	0.877	0.73	4.706
CH000004657	Nrf2 ---> HMOX1	0.903	0.62	0.952	0.877	1.328	4.68
CH000000822	c-Jun degradation	0.589	1.447	0.613	0.848	1.179	4.676
CH000004419	estradiol ---> E2F1	0.498	0.666	1.065	0.986	1.42	4.635
CH000000740	IFNalpha/beta pathway	0.72	1.337	1.093	0.639	0.826	4.615
CH000000937	PRL ---Src,FAK1---> ERK	1.079	0.543	0.839	1.414	0.721	4.596
CH000004478	IL-3 ---> c-CBL, PI3K	0.262	0.836	1.531	1.399	0.531	4.56
CH000000288	CH000000288	1.52	0.836	0.664	0.634	0.904	4.557
CH000003555	NIPA ---> cyclin B1	0.589	1.393	0.613	0.814	1.141	4.551
CH000000657	insulin ---IRS---> PRK2, SHP-2, MAPK cascade	0.655	0.682	1.596	0.634	0.806	4.372
CH000000990	p300 ---> ER	0.542	0	1.171	1.122	1.487	4.322
CH000000320	CH000000320	0	0.767	1.334	1.292	0.837	4.23
CH000000038	insulin ---> ERK	0.644	0.274	1.529	0.976	0.792	4.216
CH000003940	NMDA receptor signaling	0.903	0.154	1.334	1.162	0.623	4.176
CH000000864	Htt degradation	0.589	1.393	0.613	0.814	0.765	4.174
CH000001028	IGF-1 ---> Akt-1 ---> AR	0.589	1.642	0.613	1.292	0	4.136
CH000000760	IGF-1 pathway	0.589	1.488	1.068	0.986	0	4.131
CH000000607	BCR ---> ERK	0.709	0.188	1.529	0.986	0.695	4.107
CH000004628	TGFbeta1 ---Smad7, TAK1---> p38	0.522	0.767	0.851	0.475	1.41	4.025
CH000000438	CH000000438	0.589	0.767	1.334	1.292	0	3.982
CH000000994	EGF ---> actin polymerization	0.903	0.792	0.954	1.327	0	3.976
CH000000250	CH000000250	1.526	0.62	0.462	0.427	0.73	3.766
CH000000249	CH000000249	1.526	0.62	0.462	0.427	0.73	3.766
CH000000032	SP ---> Bcl-2	0.814	0	0.851	1.399	0.695	3.759

CH000000262	CH000000262	0.644	0	0.664	1.514	0.904	3.726
CH000000260	CH000000260	0.644	0	0.664	1.514	0.904	3.726
CH000000461	TGFbeta1 ---> Smad1, Smad2, Smad5	0.45	0	1.621	0.877	0.73	3.677
CH000000169	CH000000169	0.589	0	1.334	0.567	0.837	3.327
CH000000151	CH000000151	0.589	0	1.334	0.567	0.837	3.327
CH000000498	MKP-1 ---/ MBP	0.589	0	1.334	1.292	0	3.215
CH000004631	SMAD7, SIK1 gene induction	0.589	0	1.334	0.567	0	2.49
CH000000670	Trx1 ---> HIF-1alpha	0.498	0	0	1.729	0	2.227

Supplementary Table S3

Table 3. Medians of observed differences in pathway genes matched by pairs of patient subnetworks. The relative quantity was calculated as the number of pathway genes not shared by subnetworks divided by the number of pathway genes matched by both subnetworks combined. Calculations were performed for all pairs of patients and the 33 largest of 278 significant pathways with at least 10 pathway genes.

Pathway	Name	Median difference
CH000000770	beta-catenin network	0.800
CH000000926	PRL pathway	0.500
CH000004648	angiotensin	0.500
CH000000711	TGFbeta pathway	0.462
CH000000736	PDGF pathway	0.429
CH000000750	insulin pathway	0.429
CH000004572	EGF ---Raf-1---> ERK2	0.429
CH000004475	IL-3 signaling	0.400
CH000004506	IL-5 pathway	0.400
CH000000772	TNF-alpha pathway	0.375
CH000000722	EGF pathway	0.364
CH000000710	p53 pathway	0.333
CH000000743	tuberin pathway	0.333
CH000000755	TLR4 pathway	0.333
CH000000758	stress-associated pathways	0.333
CH000000851	JNK pathway	0.333
CH000003912	neurotrophic signaling	0.333
CH000004149	Nrf2 pathway	0.333
CH000000715	HIF-1alpha pathway	0.286
CH000000751	T-cell antigen receptor pathway	0.286
CH000000879	Caspase network	0.286
CH000004521	TLR2-mediated signaling	0.286
CH000000747	E2F network	0.273
CH000004191	ErbB3 ---> survival	0.273
CH000000947	parkin associated pathways	0.250
CH000001016	AR pathway	0.250
CH000004526	autophagy	0.250
CH000004600	mammalian Hippo network	0.250
CH000000692	S phase (Cdk2)	0.200

CH000000694	G2/M phase (cyclin B:Cdk1)	0.200
CH000004525	diacyl lipopeptide, TLR2	0.200
CH000000752	Rac1 pathway	0.167
CH000004341	ER-alpha pathway	0.143
